# Characterization of Salivary Microbiota in Japanese Patients with Oral Cancer

**DOI:** 10.3390/ijms26052339

**Published:** 2025-03-06

**Authors:** Kenichi Kumagai, Shigeo Ishikawa, Mitsuyoshi Iino, Kaoru Edamatsu, Naoki Okuyama, Kazuyuki Yusa, Yudai Shimizu, Reo Aoki, Chieko Masuda, Yoshihiro Ohashi, Akihisa Horie, Kazuto Hoshi, Yoshiki Hamada

**Affiliations:** 1 Department of Oral-Maxillofacial Surgery and Orthodontics, The University of Tokyo Hospital, Tokyo 113-8655, Japan; hoshi-ora@h.u-tokyo.ac.jp; 2 Department of Dentistry, Oral and Maxillofacial Plastic and Reconstructive Surgery, Faculty of Medicine, Yamagata University, 2-2-2 Iida-Nishi, Yamagata 990-9585, Japan; 3 Department of Oral and Maxillofacial Surgery, Shinjo Tokushukai Hospital, 1-1, 4623, Shinjo, Yamagata 996-0041, Japan; 4 Department of Oral and Maxillofacial Surgery, Kanto Rosai Hospital, 1-1, Kizukisumiyoshi, Nakahara-ku, Kawasaki 211-8510, Japan; rrlmg850@yahoo.co.jp (Y.S.); r.aoki.oms@gmail.com (R.A.); gaulchie2222@gmail.com (C.M.); y.ohashi@kantoh.johas.go.jp (Y.O.);; 5 Department of Oral and Maxillofacial Surgery, School of Dental Medicine, Tsurumi University, 2-1-3 Tsurumi, Tsurumi-ku, Yokohama 230-8501, Japan; hamada-y@tsurumi-u.ac.jp

**Keywords:** oral cancer, oral squamous cell carcinoma, salivary microbiome, 16S rRNA, high-throughput sequencing

## Abstract

This study aimed to characterize salivary microbiota in patients with oral cancer using 16S rRNA amplicon sequencing. DNA was extracted from saliva samples of 23 patients with oral cancer and 95 age-matched controls. A metagenomic analysis was performed using 16S rRNA amplicon sequencing. Patients with oral cancer exhibited lower α-diversity, as indicated by the Chao-1 index, compared to the control group, and significant differences in β-diversity were observed between the two groups. At the genus level, 25 bacterial species such as *Lautropia*, *Megasphaera*, *Lactobacillus*, *Kingella*, *Gemella*, *Staphylococcus*, and *Propionibacterium* were identified in patients with oral cancer, with more than half being Gram-positive facultative anaerobes or anaerobes. The reduced bacterial diversity in saliva of patients with oral cancer suggests dysbiosis during oral carcinogenesis may contribute to changes in bacterial distribution within the oral cavity.

## 1. Introduction

Oral cancer remains a significant global health concern, with squamous cell carcinoma arising from the oral mucosa accounting for over 90% of cases. In Japan, oral squamous cell carcinoma (OSCC) represents 1–2% of all cancer diagnoses, and its incidence is steadily increasing [1]. Despite advancements in cancer therapy, the overall 5-year survival rate for patients with OSCC has plateaued at approximately 50% over recent decades [2]. However, early detection markedly improves survival prospects, increasing the rate to approximately 90% [3,4]. While most OSCC cases are macroscopically detectable, a substantial proportion of patients still present with advanced-stage disease. Therefore, the early detection of OSCC is crucial for improving the postoperative quality of life (QOL) and prognosis. Consequently, there is an urgent need for the development of novel and highly reliable diagnostic tools for OSCC.

The oral cavity is second only to the gut in the size and diversity of its microbial community, harboring over 700 bacterial species. Recent research has highlighted the role of the gut microbiome in various diseases, with next-generation sequencing (NGS) offering a powerful approach for their detection [5]. Furthermore, distinctive oral microbiome profiles have been reported in patients with gastric and pancreatic cancers [6,7]. However, investigations of the oral microbiota in patients with OSCC and individuals with precancerous lesions have been limited in scope, and their findings have often been inconsistent [8,9,10]. Conversely, the detection of oral commensal bacteria in metastatic cervical lymph nodes of patients with OSCC suggests a potential link between the oral microbiome and OSCC progression. If oral microbiome analysis could be clinically translated for OSCC detection, novel cancer control strategies could be developed, potentially including OSCC prevention through the targeted use of probiotics. Over the past decade, there has been growing interest in the potential association between the oral microbiome and various stages of cancer development. However, the relationship between the oral microbiome and oral cavity cancer remains incompletely understood, as a comprehensive characterization of oral microbiome profiles in Japanese healthy controls and patients with OSCC is lacking.

In this study, we investigated the salivary microbiome of Japanese patients diagnosed with oral cancer using 16S rRNA amplicon sequencing. We compared these findings to those from age-matched healthy donors to assess the feasibility of using oral microbiome profiles as a novel diagnostic tool for OSCC.

## 2. Results

### 2.1. Diversity of Oral Flora

**Figure 1:**
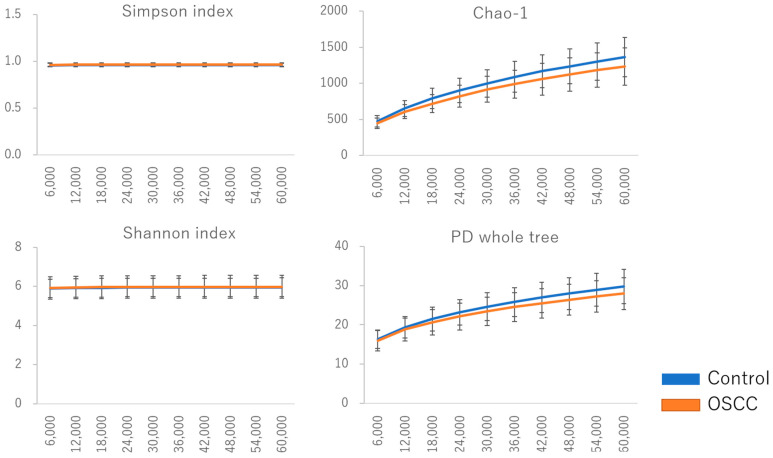
Diversity of oral flora in saliva among controls and patients with oral cancer. Various indexes (Simpson, Chao 1, Shannon, and PD whole tree) were utilized to assess the α-diversity of the bacterial community. The diversity of the bacterial community in OSCC samples was significantly lower compared to controls, as indicated by the OTU observed and Chao 1 index (*p* < 0.05). No significant differences were observed with the Shannon and Simpson indices.

**Figure 2:**
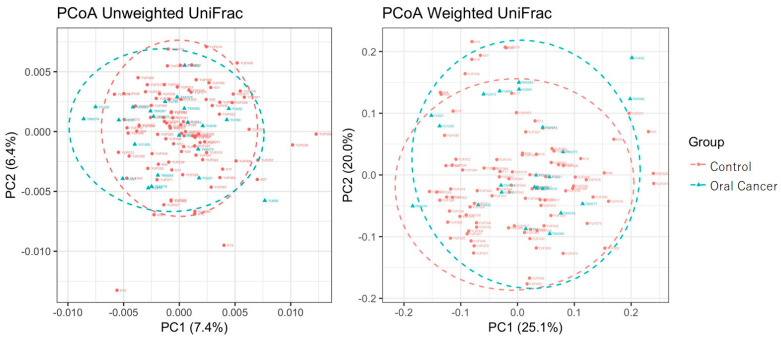
Principal coordinate analysis (PCoA) on weighted and unweighted UniFrac distances in controls and patients with oral cancer. The Pseudo-F statistics for unweighted and weighted UniFrac distances were 2.1047 and 2.1602, respectively. The *p*-values for unweighted and weighted UniFrac distances were 0.001 and 0.048.

The characteristics of the control group and patients with oral cancer in this study are presented in Table 1. Various alpha diversity indices (Simpson, Chao1, Shannon, and Phylogenetic Diversity [PD] whole tree) were employed to assess the diversity of the bacterial communities. A total of 9255 operational taxonomic units (OTUs) were identified, with 8854 observed in the control group and 5359 in patients with oral cancer. Four thousand nine hundred and fifty-eight (4958) OTUs were shared between the two groups, suggesting a reduction in bacterial diversity in patients with oral cancer (Table 2). The Chao1 index indicated a significantly lower diversity in the bacterial communities of OSCC samples compared to controls (*p* < 0.05). No significant differences were observed for other diversity indices, such as Shannon and Simpson (Figure 1). To assess overall differences in bacterial taxa composition, a principal coordinate analysis (PCoA) was performed based on weighted and unweighted UniFrac distances. Statistically significant differences were observed in both weighted (*p* = 0.009) and unweighted (*p* = 0.003) UniFrac distances between OSCC samples and controls (Figure 2).

### 2.2. Relative Abundance of Oral Flora

**Figure 3:**
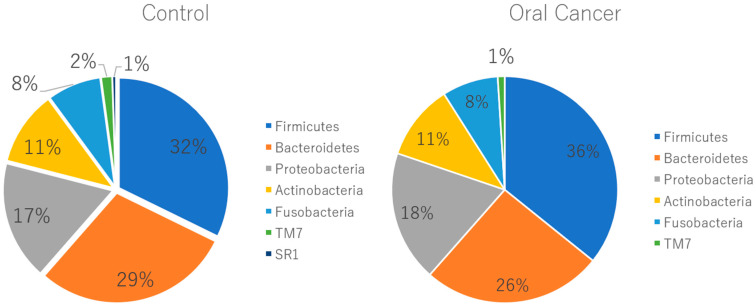
Relative abundance of oral flora at the phylum level. At the phylum level, there was no significant difference in percentage between healthy subjects and patients with oral cancer, except for *Saccharibacteria*, which showed a higher abundance in healthy subjects (*p* = 0.04). *Chlorobi* and *Elusimicrobia* were exclusively found in patients with oral cancer, though their relative amounts were less than 0.1%.

**Figure 4:**
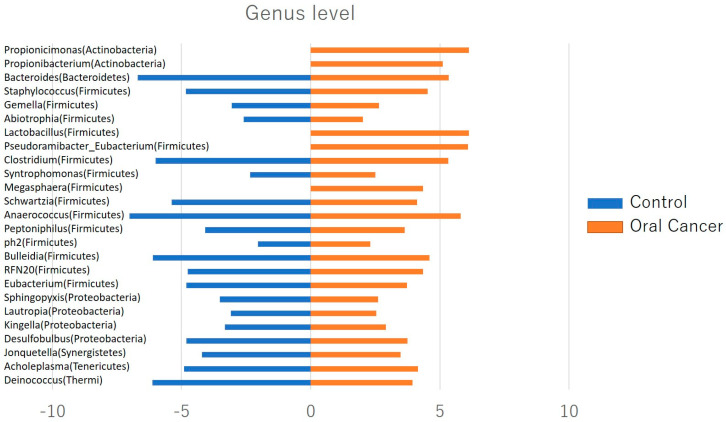
Relative abundance of oral flora at the genus level. Twenty−five bacterial species, including *Lautropia*, *Megasphaera*, *Lactobacillus*, *Kingella*, *Gemella*, *Staphylococcus*, and *Propionibacterium*, were identified in patients with oral cancer, with more than half of them being Gram-positive facultative anaerobic to anaerobic bacteria.

At the phylum level, Firmicutes, Bacteroidetes, Proteobacteria, Actinobacteria, Fusobacteria, and *Saccharibacteria* were the predominant taxa in the salivary microbiota of both patients with OSCC and controls. *Saccharibacteria* was significantly more abundant in patients with oral cancer compared to controls (Figure 3). At the genus level, twenty-five bacterial genera, including Lautropia, Megasphaera, Lactobacillus, Kingella, Gemella, Staphylococcus, and Propionibacterium, were identified in patients with oral cancer. Over half of these were Gram-positive facultative anaerobes or anaerobes (Figure 4).

### 2.3. TNM Classification

**Figure 5:**
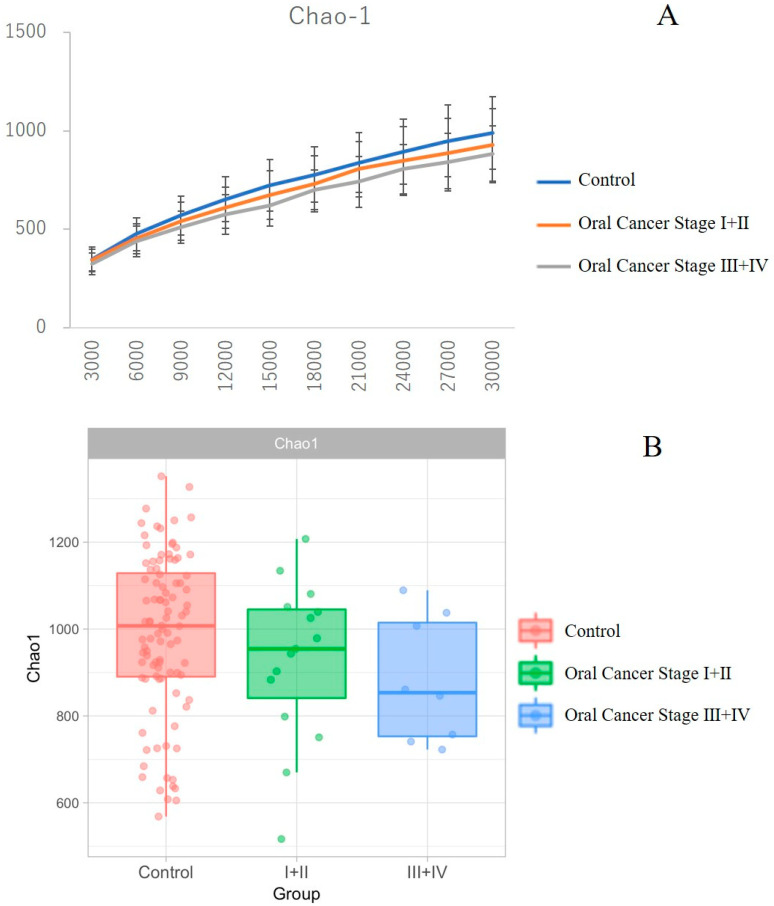
Comparison of diversity across clinical stages in patients with oral cancer. Panel B shows the box-and-whisker plot. There was a trend of decreasing α-diversity as measured by Chao-1 with advancing clinical stage; however, no significant differences were observed between the control group, oral cancer stage I + II groups, and stage III + IV groups (**A**,**B**).

Stratified analysis was performed to investigate the association between tumor stage and the relative abundance of dominant taxa using the TNM (tumor–node–metastasis) classification system of the International Union Against Cancer (UICC) (7th edition). While differences in OTUs were observed between patients with oral cancer and controls, T-stage progression, as defined by the TNM classification, did not exhibit a significant association with microbial composition. We observed a trend towards decreasing alpha diversity, as measured by Chao1, with increasing clinical stage. However, differences between the control group, OSCC stages 1 and 2 combined, and stages 3 and 4 combined, were not statistically significant (Figure 5A,B).

## 3. Discussion

In this study, we investigated the association between oral microorganisms and oral cancer, revealing a reduced diversity of the salivary microbiome in patients with OSCC. Several studies have employed next-generation sequencing (NGS) to characterize the oral microbiota associated with oral cancer [11,12,13]. Previous studies have suggested such an association. However, these investigations have been limited by small sample sizes and the use of sample tissues from both cancer lesions and healthy sites within the same subject.

An established relationship exists between the gut microbiota and tumorigenesis in the digestive tract. Notably, *Helicobacter pylori* has now been recognized as a carcinogenic agent in gastric cancer [14], and low grade B-cell MALT gastric lymphoma initiation [15]. *Salmonella typhi* and *Fusobacterium* are associated with gallbladder and colon cancer, respectively [16,17]. Furthermore, a potential association between the oral microbiota and colorectal cancer has been reported, with several oral taxa, such as *Streptococcus* and *Prevotella* were found to be abundant in colorectal cancer patients compared to a healthy control group [18].

Saliva, being more homogeneous than tissue, is a preferable and non-invasive biosource for microbiome analysis. At the genus level, 25 species of bacteria such as *Lautropia*, *Megasphaera*, *Lactobacillus*, *Kingella*, *Gemella*, *Staphylococcus*, and *Propionibacterium* were found in patients with oral cancer, more than half of which were Gram-positive facultative anaerobic or an anaerobic bacterium. It was considered that the Gram-positive facultative anaerobic or an anaerobic bacterium that increased predominantly in patients with OSCC could serve as salivary biomarkers of oral cancer. Currently, serum markers for OSCC are only supplementary used and do not always reflect the tumor clinical condition. Therefore, they are not suitable for OSCC screening. Regarding the easiness of sample collection, saliva has thought to be more suitable for screening compared with blood. Saliva is a promising specimen for investigations of the oral environment. Otherwise, a role for bacterial infection in causing or promoting cancer is well known with respect to the association of *Helicobacter pylori* with gastric cancer [19], and other cancers, including gallbladder, colon, lung and prostate, have been associated with particular bacterial infections [20,21,22].

The microbial environment in the human body plays an essential role in maintaining health through interactions with nutrient absorption, the immune system, and various metabolic processes. Furthermore, host–microbe interactions counteract invading pathogens and prevent tumorigenesis [23]. However, disruptions in the microbiota composition can shift homeostasis towards dysbiosis. Such imbalances in the local microbial environment could modulate the host’s immune responses and inflammation, thereby favoring disease pathogenesis and progression [24]. Previous studies raised questions regarding the role of the oral microbiome in the progression of oral cancer and whether microbiome change is a significant risk factor in the development of oral cancer [25,26]; however, a large-scale comparison of the oral microbiota between Japanese patients with oral cancer and non-cancer control groups has been lacking, and therefore, the credibility of the findings of oral microbiome profiles in OSCC is unclear.

Due to the limited sample size and limitations in the differential abundance analysis methods tailored for microbiome data, it is difficult to definitively assess the feasibility of oral microbiome profiles as a novel detection tool for OSCC; however, our results demonstrate reduced diversity in patients with OSCC. The oral microbiome typically exists in the form of a biofilm, playing a crucial role in maintaining oral homeostasis, protecting the oral cavity, and preventing disease development. Alterations in the oral microbiome in tumors compared to controls suggest that changes in oral community structure may result in alterations in functional pathways with systemic relevance.

## 4. Materials and Methods

### 4.1. Sample Collection

#### Subjects

We enrolled 23 patients diagnosed with oral cancer (17 men, 6 women; mean age 65.8 years, range 31 to 87 years) who underwent surgery at Tsurumi University and Kanto Rosai Hospital from May 2017 to December 2019. Age-matched controls in Yamagata University Hospital, and Tsurumi University were individuals over 25 years of age without diagnosed mucosal diseases or other cancers. All oral cancer diagnoses were confirmed as squamous cell carcinoma via biopsy and pathological examination. Participants failing to comply with instructions were excluded. All patients with oral cancer and age-matched controls completed a questionnaire on gender, age, alcohol consumption, and smoking habits.

### 4.2. Sample Collection Time

A total of 119 salivary samples from patients were included in the study. Participants collected unstimulated saliva samples for 5 min in sterile plastic tubes, stored at −80 °C until use. Saliva from patients with oral cancer was collected one week pre-operation, and subsequently at 1 to 2 h intervals post-tooth brushing and meal consumption. Antibiotics were not administered for at least one week prior to saliva collection to ensure accurate representation of the microbiome.

### 4.3. DNA Extraction

Microbial DNA was extracted from saliva samples using the Isospin Fecal DNA Kit (NIPPON GENE Co., Ltd., Tokyo, Japan) following a standard protocol. Quantity and quality of isolated DNA were assessed using a Qubit 3.0 Fluorometer (Thermo Fisher Scientific, Waltham, MA, USA) and agarose gel electrophoresis, respectively. DNA samples were stored at −20 °C for further analysis.

### 4.4. 16S rRNA Sequencing

The V1-V2 regions of the 16S rRNA genes were amplified and sequenced on the MiSeq Deep sequencer using MiSeq Reagent Kit v3 (Illumina, San Diego, CA, USA), following the manufacturer’s protocol. Amplicon libraries from individual samples were generated by PCR amplification using primers 27F (5′-AGRGTTTGATYMTGGCTCAG-3′) and 338R (5′-TGCTGCCTCCCGTAGGAGT-3′) targeting the V1-V2 hypervariable region of the 16S rRNA gene. PCR conditions included an initial denaturation at 95 °C for 3 min, followed by 25 cycles at 95 °C for 30 s, 55 °C for 30 s, 72 °C for 30 s, and a final extension at 72 °C for 5 min. PCR products were purified using 15 μL of Agencourt AMPure XP (Beckman Coulter, Inc., Brea, CA, USA) as per the manufacturer’s protocol. Illumina adapters were attached using the Illumina MiSeq Nextera kit set A (Illumina Inc., San Diego, CA, USA). Sequencing was performed on the Illumina MiSeq platform following the manufacturer’s instructions.

### 4.5. Bioinformatics Analysis

FASTQ files were obtained after Illumina paired-end 16S rRNA gene amplicon sequencing. Operational taxonomic unit (OTU) classification and diversity analyses were conducted using QIIME version 1.9.1 [14], following established methods [15]. OTU picking employed the open-reference method with a 97% identity pre-filtered Greengenes database and uclust. Representative sequences of each OTU were selected, and taxonomy assignment was performed using the RDP classifier with a threshold score of 0.5 or higher. OTUs were grouped based on identical annotations regardless of RDP score.

### 4.6. Statistical Analysis

Data were exported as BIOM files and analyzed in R (version 3.5.0). A diversity analysis utilized the “phyloseq” R package [18]. Alpha-diversity indexes (Simpson Index, Chao1 Index, Shannon Index, and PD whole tree) were calculated using the estimate richness function, while beta-diversity indices (unweighted UniFrac distance and weighted UniFrac distance) were generated using the unifrac function in “phyloseq”. A principal coordinate analysis (PCoA) assessed similarity in oral bacterial community structure among samples using the dudi.pco function in the “ade4” R package. Dominant bacteria from phylum to genus level were defined as the mean distribution with at least 0.05% abundance. Student’s *t*-test compared dominant oral bacterial communities (>1% abundance) between oral cancer (OC) and healthy controls (HCs) using the t.test function in the “tool” R package. To compare taxon abundances among OC samples of different T-stages, Wilcoxon rank sum tests and Pearson correlation analyses were conducted using the “tool” R package. All statistical tests were two-sided, with significance set at *p* < 0.05. Graphs were created using the “ggplot2” R package [19], and data are presented as means ± SD. Differences between groups were assessed using Student’s *t*-test, with *p* < 0.05 indicating significance.

## 5. Conclusions

The reduced bacterial diversity in saliva of patients with oral cancer suggests the dysbiosis during oral carcinogenesis may contribute to alterations in the distribution of specific bacterial taxa within the oral cavity.

## Figures and Tables

**Table 1:** Characterization of controls and patients with oral cancer in this study.

		Control	Oral Cancer
Total number		95	23
Male (%)		69.5	56.5
Female (%)		30.5	43.5
Age, mean		61.8	65.8
Clinical Stage (n)			
	Ⅰ + Ⅱ		16
	Ⅲ + Ⅳ		7
primary site (n)			
	Gingiva		11
	Tongue		8
	Buccal mucosa		2
	Maxillary		1
	Mandibular		1
Smoker (n)		-	3
non-Smoker (n)		-	20
Alchole (n)		-	11
non-Alchole (n)		-	12

**Table 2:** OUT observation of controls and patients with oral cancer in this study.

	Control	Oral Cancer
Total OUT	8854	5359
ave. ± S.D.	885.0 ± 191.1	957.2 ± 135.2

## Data Availability

Data are contained within the article.
